# Resistance to Antimalarial Monotherapy Is Cyclic

**DOI:** 10.3390/jcm11030781

**Published:** 2022-01-31

**Authors:** Rachel Weitzman, Ortal Calfon-Peretz, Trishna Saha, Naamah Bloch, Karin Ben Zaken, Avi Rosenfeld, Moshe Amitay, Abraham O. Samson

**Affiliations:** 1 Bioinformatic Department, Jerusalem College of Technology, Jerusalem 9372115, Israel; w.rachel99@gmail.com (R.W.); ortal.calfon@gmail.com (O.C.-P.); mosh9900@gmail.com (M.A.); 2 Drug Discovery Lab, Azrieli Faculty of Medicine, Bar Ilan University, Safed 1311502, Israel; trishna.saha27@gmail.com (T.S.); naamah.bloch@biu.ac.il (N.B.); karinab1992@gmail.com (K.B.Z.); 3 Department of Computer Science, Jerusalem College of Technology, Jerusalem 9372115, Israel; rosenfa@jct.ac.il

**Keywords:** malaria, drug resistance, antimalarial resistance, text-mining, parasitology, health policy, drug of choice, *Plasmodium falciparum*, *Plasmodium vivax*, *Plasmodium*, antibiotic resistance

## Abstract

Malaria is a prevalent parasitic disease that is estimated to kill between one and two million people—mostly children—every year. Here, we query PubMed for malaria drug resistance and plot the yearly citations of 14 common antimalarials. Remarkably, most antimalarial drugs display cyclic resistance patterns, rising and falling over four decades. The antimalarial drugs that exhibit cyclic resistance are quinine, chloroquine, mefloquine, amodiaquine, artesunate, artemether, sulfadoxine, doxycycline, halofantrine, piperaquine, pyrimethamine, atovaquone, artemisinin, and dihydroartemisinin. Exceptionally, the resistance of the two latter drugs can also correlate with a linear rise. Our predicted antimalarial drug resistance is consistent with clinical data reported by the Worldwide Antimalarial Resistance Network (WWARN) and validates our methodology. Notably, the cyclical resistance suggests that most antimalarial drugs are sustainable in the end. Furthermore, cyclic resistance is clinically relevant and discourages routine monotherapy, in particular, while resistance is on the rise. Finally, cyclic resistance encourages the combination of antimalarial drugs at distinct phases of resistance.

## 1. Introduction

Malaria is a pernicious parasitic infection transmitted by anopheline mosquitoes. Four species of Plasmodium commonly infect humans, but only one, *Plasmodium falciparum* (*P. falciparum*), accounts for most cases of morbidity and mortality [1]. Disturbingly, drug resistance has emerged in all classes of antimalarials, and it is responsible for considerable malaria-related mortality, particularly on the African continent. Drug resistance is partially alleviated through combination therapy of antimalarials, and Artemisinin-based combination therapies (ACTs) have been recommended by the World Health Organization (WHO) as a first-line treatment for *P. falciparum* malaria in countries where resistance has compromised the efficacy of other drugs [2]. As a result, resistance to ACTs has increased in western Cambodia, which could be disastrous for global malaria control [3]. According to the WWARN website [4], this trend is continuing, and resistance to ACTs has grown worldwide. Importantly, drug resistance is a survival mechanism through which the Plasmodium parasite endures, and several drug resistance mechanisms have been reported [5].

Antimalarial drugs act through various mechanisms and at different stages of the Plasmodium life cycle [6]. Specifically, quinine, chloroquine, amodiaquine, mefloquine, piperaquine [7], and halofantrine interfere with the biocrystallization of heme, thus leading to toxic heme buildup, which disrupts membrane function and results in cell lysis [8]. Likewise, artemsinin [9], artesunate [10], and artemether [11], which are prodrugs of dihydroartemisinin, bind to hemozoin and release toxic free radicals. In addition, mefloquine has recently been suggested to target the 80S ribosome of *P. falciparum*, thus inhibiting protein synthesis and leading to schizonticidal effects [12]. Doxycycline inhibits the synthesis of nucleotides and deoxynucleotides in *P. falciparum* [13], while sulfadoxine and pyrimethamine inhibit folate synthesis [14]. Atovaquone is a competitive inhibitor of ubiquinol, and specifically inhibits the mitochondrial electron transport chain [15]. Notably, several additional mechanisms of action have been proposed for these antimalarials. For example, quinine also targets the purine nucleoside phosphorylase of *P. Falciparum* [16].

Antibiotic drug resistance has been associated with periodic patterns [17]. Over a period of 10 years, antibiotic resistance of *Pseudomonas aeruginosa* has risen and fallen at a single university hospital center in Germany [18]. Likewise, yearly trends of meropenem antimicrobial resistance oscillate in 10-year intervals in the US [19]. The antibiotic resistance of ocular micro-organisms also follows a similar trend, and drug resistance is periodic [20]. Publications by the Center for Disease Control and Prevention, also illustrate these ebb and flow patterns, and antibiotic resistance threats are periodic (www.cdc.gov/drugresistance/pdf/ar-threats-2013-508.pdf, accessed on 27 May 2021).

Text mining, citation counts, and frequency analyses are often used to identify trends and patterns in medicine. Several studies have used text mining, and notably, Bork et al. have captured the phenotypic effects of drugs based on the side effects resources published by the FDA [21]. In another study, Jensen and coworkers have used text mining to associate diseases and genes and to establish a web-based database named DISEASE [22]. In the past, we have used frequency analyses of PubMed citations to show that antibiotic resistance is periodic [17]. Here, we use PubMed citations to evaluate the periodicity of drug resistance to antimalarial monotherapy and validate our findings using clinical data from the WWARN [4].

## 2. Methods

*PubMed text mining.* To evaluate malaria drug resistance, we counted the yearly occurrence of the word combinations of ‘drug’, ‘malaria’, and ‘resistance’ in the scientific literature cited on PubMed. For example, for chloroquine malaria resistance in 1986, we counted the number of citations containing “chloroquine AND malaria AND resistance” in the title or abstract, as indexed by PubMed, in that year. Then, we normalized our data by dividing it by the number of papers containing either “chloroquine OR malaria OR resistance”. We note that this method captures noise (i.e., false citations) in addition to signal (i.e., true citations); the signal to noise ratio (SNR) is expected to increase with the number of citations, and noise is expected to decrease with normalization.

We performed this procedure for some 30 generic antimalarial drugs (i.e., quinine, quinidine, chloroquine, hydroxychloroquine, amodiaquine, atovaquone, primaquine, mefloquine, tafenoquine, artemether, artemisinin, artesunate, dihydroartemisinin, sulfadoxine, halofantrine, lumefantrine, piperaquine, pyrimethamine, chlorproguanil, etc.) and prophylactic agents (i.e., azithromycin, doxycycline, clindamycin, trimethoprim, proton pump inhibitor, H2 blocker, vitamin B, etc.) for every year between 1980 and 2020. Finally, after applying a smoothing function of a 3-year average, we fitted the normalized citations corresponding to drug resistance to a periodic function and calculated correlations using Excel.

*World antimalarial resistance network mining.* To evaluate the malaria drug resistance in clinical settings, we retrieved the “lowest efficacy rate (%)” of monotherapy for all locations on the World Antimalarial Resistance Network website (WWARN, http://www.wwarn.org/explorer/app, accessed on 1 October 2021) [4]. The monotherapy drug resistance (%) at each location was calculated using the following formula:

[Drug Resistance] (%) = 100 − [Lowest Efficacy Rate] (%)

The average yearly resistance was calculated as the average resistance of all locations in a given year, and a smoothing function of a 3-year average was applied. The average early monotherapy resistance was readily available for quinine, amodiaquine, and chloroquine.

*Experimental validation.* The scientific literature was searched for experimental trends of antimalarial drug resistance, and the reported yearly experimental resistance was plotted as the percentage of resistance. For Singhasvanon [23], the yearly resistance (%) of quinine and mefloquine were copied. For Frosch [24], the yearly resistance (% prevalence of the chloroquine resistance marker, Pfcrt 76T) was taken as the average of the 8 African countries reported, namely Niger, Uganda, Mali, Tanzania, Guinea Bissau, Malawi, Burkina Faso, and Kenya.

## 3. Results

*Antimalarial drug resistance is cyclic.* Of the 30 generic antimalarial drugs, only 12 presented an average count of 20+ yearly citations over the past 40 years, which was deemed necessary for statistical significance (i.e., quinine, chloroquine, mefloquine, amodiaquine, artesunate, artemisinin, dihydroartemisinin, artemether, sulfadoxine, doxycycline, halofantrine, piperaquine, atovaquone, and pyrimethamine). Notably, 2 more drugs are also included in our analysis even though they presented <20 citations in some years (i.e., doxycycline and piperaquine). The other 16 drugs were discarded from our analysis, as they did not present 20 yearly citations. 

**Figure 1:**
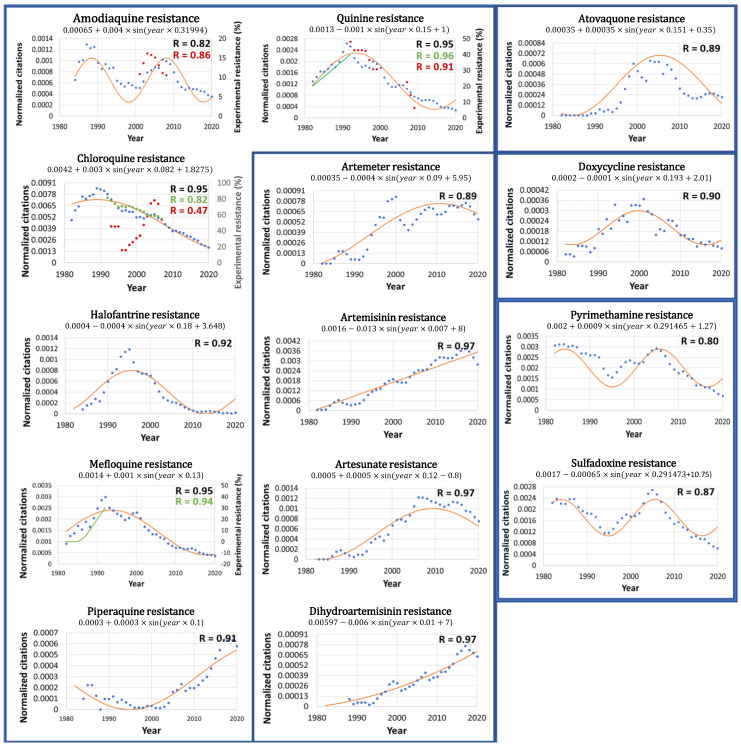
Antimalarial drug resistance is cyclic. The resistance estimated through PubMed citations (blue dots) and the fitted periodic function (red line) are plotted. The fitted equation is listed above each graph, as is the correlation coefficient, R (in black). Notably, antimalarial drug resistance correlates well with sine functions. Artemisinin and dihydroartemisinin also correlate with a linear rise. The clinical monotherapy resistance reported by WWARN 4 is also plotted (red dots), as is the correlation with the PubMed citations, R (in red). Finally, experimental resistance reported by Singhasivanon23 (green line) and Frosch25 (green dots) is plotted, as is the correlation with PubMed citations, R (in green). Notably, the resistance estimated by the normalized PubMed citations correlates well with clinical and experimental data and validates our methodology.

Remarkably, the normalized citations display cyclic patterns (Figure 1), and antimalarial drug resistance has been fitted to a sine function using the following equation:

[Drug resistance] = Constant + Multiplier * sin (Year * 2π [Frequency] + [Phase])

For each antimalarial drug, the frequency and phase have been calculated using fast Fourier transform, and the curve has been fitted using the least square deviation method. The frequency and phase are listed above each drug graph. Based on these values, antimalarial drug resistance may be projected into the future, and peaks and troughs may be predicted. 

Notably, the predicted resistance of the antimalarial drugs correlates well with cyclic sine functions (R > 0.8, *p* < 0.05). The antimalarial drugs that exhibit cyclic resistance are quinine (R = 0.95), chloroquine (R = 0.95), mefloquine (R = 0.95), amodiaquine (R = 0.82), artesunate (R = 0.97), artemisinin (R = 0.97), dihydroartemisinin (R = 0.97), artemether (R = 0.89), sulfadoxine (R = 0.87), doxycycline (R = 0.90), halofantrine (R = 0.92), piperaquine (R = 0.91), atovaquone (R = 0.89), and pyrimethamine (R = 0.8). Exceptionally, the resistance of artemisinin and dihydroartemisinin can also fit with a linear rise (R > 0.9). The continuous rise of artemisinin resistance, based on PubMed citations and shown in Figure 1, agrees with a gradual increase in resistance to artemisinin and artemisinin-based combination therapies (ACT) worldwide [25]. Notably, one of the main contributions to the continuous rise of global artemisinin resistance could also be a direct result of the WHO recommendation to use ACTs to treat malaria.

*Experimental validation.* Remarkably, our global resistance patterns of amodiaquine, quinine, and chloroquine are validated by local clinical trials reported on the WWARN website (Figure 1, red dots) [4]. For example, the correlation between the global resistance and the average of several local clinical resistances is significant, as indicated by the high correlation coefficients of quinine (R = 0.91) and amodiaquine (R = 0.86, upon a 4-year shift, and R = 0.76 upon a 3-year shift). The correlation of global and local resistance is high if several local measurements are averaged. Conversely, the same correlation is low if few local measurements are available. For example, the correlation coefficient of chloroquine (R = 0.47) is low, and is due to great local variations in WWARN and single data points. For instance, in 2001, WWARN reported 16% chloroquine resistance based on 2 clinical trials with a 40% standard deviation, while in 2004, it reported 93% resistance based on one single clinical trial. If the single trial is ignored, then the chloroquine correlation rises to R = 0.64. If the 2 clinical trials with a 40% standard deviation are ignored, then the chloroquine resistance correlation rises to R = 0.96. Thus, our global resistance patterns are not synchronous with the single-point local data of WWARN, nor with two-point data with large standard variations. Our global resistance patterns are synchronous with averaged WWARN data points sampled from multiple locations.

Interestingly, our predicted resistance of quinine and mefloquine is validated by earlier reports of experimentally observed resistance (Figure 1, green line) [23]. The correlation between the experimental and predicted resistances is significant, as indicated by the correlation coefficients (R) of quinine (0.96) and mefloquine (0.94). Our findings are also validated by average yearly chloroquine resistance patterns (R = 0.82) reported by Frosch [24], which on average, declined in several countries of Africa after 1992 (Figure 1, green dots). Notably, the prevalence of chloroquine resistance varies greatly between African countries, yet the average shows a gradual decline since 1992. It is also interesting to note that the average African chloroquine resistance correlates highly with that reported for French Guiana (R = 0.92, data not shown) [26]. As such, this South American region could potentially serve as an indicator of global chloroquine resistance. Our findings are also in agreement with the global rise of artemisinin resistance as reported for several countries of Asia [27,28] and the world [25]. Notably, yearly global resistance patterns are scarce, yet those reported here agree with our predicted resistance patterns shown in Figure 1.

In addition, our predicted resistance patterns are in agreement with molecular resistance markers measured over several years [29]. For example, our predicted fall of chloroquine resistance between 2003 and 2015 corresponds well with the decline of the allele frequency of the chloroquine resistance marker, Pfcrt 76T, from 0.7 in 2003 to 0.1 in 2015 in Kenya. Likewise, our predicted fall of sulfadoxine and pyrimethamine resistance between 2003 and 2008 correlates well with the decline of the allele frequency of the sulfadoxine–pyrimethamine resistance marker, Pfdhfr51I-59R-108N/Pfdhps437G-540E, from 0.7 in 2003 to 0.2 in 2008 in Kenya. Finally, our predicted rise of artemether resistance between 2003 and 2015 correlates well with the increase in the allele frequency of the artemether–lumefantrine resistance markers, Pfmdr1 N86, 184F, and D1246, from 0.1 in 2003 to 0.4 in 2015. Thus, our predicted resistance patterns are also validated at a molecular level. Notably, Hemming-Schroeder et al. show that the allelic frequency of resistance is governed by the timeline of antimalarial drug policy and drug use [29]. Thus, our predicted resistance patterns also correlate well with the timeline of antimalarial drug policies and drug use.

*Monotherapy is disfavored.* Our data suggest that antimalarial monotherapy should be avoided—in particular, when resistance spikes. The data also advocate the use of combination therapies other than ACT whose resistance is on the rise. The data highlight the potential efficacy of combination therapies using drugs at different phases of their resistance—for example, chloroquine, atovaquone, and sulfadoxine. Interestingly, our analysis of resistance to combination therapies does not yield statistically significant results (data not shown), perhaps because combination therapies are less susceptible to resistance.

*Antimalarial drugs with similar mechanisms of action share similar resistance cyclicity.* Interestingly, the cyclical resistance frequency may be associated with the drug mechanism of action (Table 1).

For example, pyrimethamine (frequency = 0.29) and sulfadoxine (frequency = 0.29) are both folic acid synthesis inhibitors and share identical cyclic frequencies around **0.3**. The idea that shared mechanisms of action are correlated with similar periods of resistance is not new, and has been previously proposed by us [17]. We carefully note that this similarity in frequency could also be due to their co-administration, and less to their similar mode of action.

Quinine (frequency = 0.15), chloroquine (frequency = 0.082), mefloquine (frequency = 0.13), piperaquine (frequency = 0.1), and halofantrine (frequency = 0.18) are heme biocrystallization inhibitors and share periodic frequencies around **0.15**. This range is broad and could, in fact, indicate multiple mechanisms of action, as indicated by newly discovered targets of these drugs [16]. Different mechanisms of action are expected to give rise to different periods of resistance. Notably, amodiaquine (frequency = 0.32) is an outlier, possibly due to multiple mechanisms of action [30]. We carefully note that this similarity could also result from frequent coadministration with pyrimethamine (frequency = 0.29) and sulfadoxine (frequency = 0.29), as mentioned above. As a result, amodiaquine is not listed in Table 1.

Artemisinin (frequency = 0.01) and its active metabolite, dihydroartemisinin (frequency = 0.01), which binds to hemozoin and releases toxic free radicals, could also fit a linear rise in resistance over the past four decades. Notably, the linear rise is also shared with artemether (frequency = 0.09) and could be due to their similar modes of action. Exceptionally, artesunate (frequency = 0.12), another related prodrug of dihydroartemisinin, plunges from a linear rise after 2005. This exception implies that artemisinin and dihydroartemisinin could also plunge from their linear rise, given the right conditions. Alternatively, artesunate’s exception also suggests more than one mechanism of action, unlike that of the artemisinins.

Atovaquone (frequency = 0.15) is a competitive inhibitor of the mitochondrial electron transport chain and shares similar periodic frequencies with mefloquine and doxycycline.

Finally, doxycycline (frequency = 0.193), and possibly mefloquine (frequency = 0.13), are protein synthesis inhibitors and share frequencies around **0.2**. Notably, the resistance curves may serve as indicators for similar mechanisms of action.

## 4. Discussion

In this study, we use PubMed citations and show that antimalarial drug resistance is correlated with a cyclical function. Initially, we hypothesized that antimalaria drug resistance is exponential, as often reported in the literature. Yet, the correlation between the yearly citations of antimalarial drug resistance and cyclic functions is higher than 0.8 and attests to the cyclicity of antimalarial drug resistance. Our correlation is validated by clinical trials and experimental data, and notably, the correlation between the yearly PubMed citations and clinical antimalarial monotherapy drug resistance is statistically significant (R coefficient > 0.8).

As shown here, most antimalarial resistance is governed by a cyclic function. This observation is clinically relevant and supports a combination therapy of two or more antimalarial drugs [31]. As single therapies fail, combination therapies (i.e., primaquine and tafenoquine) are necessary to achieve a “radical cure” and to eliminate the parasite [32].

Cyclic antimalarial drug resistance adheres to Darwin’s principle of natural selection. At first, antimalarial resistance is an uncommon and sporadic trait. Then, following worldwide use of the antimalarial drug, the survival of resistant parasites is favored, which, in turn, gives rise to resistance. Then, as resistance increases, the drug becomes less used. Finally, as drug use declines, natural selection diminishes and resistance decreases.

In addition, our data favor the use of antimalarial drugs used during the rising phase of resistance, and disfavor use once the resistance peak is reached (i.e., artemisinin and dihydroartemisinin). Finally, these data suggest that most antimalarial drugs are sustainable in the long run [33]. Importantly, these data should not deter the scientific community from developing new antimalarial drugs and reinforcing our medicinal arsenal against this nefarious disease.

As a potential limitation to this study, it should be noted that the normalized citations also correlate well with non-cyclical functions, such as polynomial functions of order 3 and up (R > 0.8), and the yearly citations of antimalarial drug resistance also fit high-order polynomial functions. Importantly, our data normalization is required in order to account for pointwise mutual information (PMI) [34]. For example, if in a given year, antimalaria drugs are overly cited, then in the same year, less weight should be given to their incidence with resistance. Likewise, if during one year, drug resistance is cited greatly, then less importance should be given to its association with malaria. Thus, normalization has been used to account for pointwise mutual information.

In addition, this study does not distinguish between the resistance of the various strains of the Plasmodium agent (*P. falciparum*, *P. vivax*, etc.) and their geographical locations, and antimalarial resistance is rendered globally uniform. Nevertheless, our theoretical predictions of antimalarial resistance correlate well with regional experimental data published by Singhasivanon [23] (Figure 1) and clinical data published by WWARN worldwide [17]. As a result, citations per year may not reflect the accurate prevalence of antimalarial resistance in local clinical settings. While costly experimental data are incomplete, and until more experimental data become available, our inexpensive theoretical data could serve as an approximate trend line for global antimalarial resistance.

## 5. Conclusions

In conclusion, antimalarial resistance, derived from PubMed citations, is cyclical. Notably, the predicted resistance is consistent with experimental and clinical data and suggests that even though most antimalarial monotherapies are sustainable in the long run, they should be avoided—particularly while resistance is high. Finally, the cyclic resistance favors combination therapies of antimalarial drugs, particularly those with low resistance.

## Figures and Tables

**Table 1:** Cyclic resistance frequency and antimalarial drug mechanism.

Antimalarial Drug	Mechanism of Action	Yearly Frequency
Pyrimethamine, Sulfadoxine	Folic acid synthesis inhibitors	~0.3
Doxycycline (prophylactic)	Protein synthesis inhibitors	~0.2
Atovaquone	Electron transport chain inhibitor	~0.15
Quinine, Chloroquine, Piperaquine, Halofantrine, Mefloquine	Heme biocrystallization inhibitors	~0.15
Artemether, Artemisinin, Dihydroartemisinin, Artesunate,	Binds to hemozoin and releases toxic free radicals	~0.05

## Data Availability

Not applicable.
